# Global transcriptomic response of *Malassezia pachydermatis* isolates under ketoconazole-induced stress

**DOI:** 10.3389/fvets.2026.1906705

**Published:** 2026-08-05

**Authors:** Marianna Domán, Enikő Wehmann, László Makrai, Enikő Fehér, Tibor Magyar

**Affiliations:** 1 HUN-REN Veterinary Medical Research Institute, Budapest, Hungary; 2 Autovakcina Kft., Budapest, Hungary; 3 Department of Microbiology and Infectious Diseases, University of Veterinary Medicine, Budapest, Hungary

**Keywords:** differentially expressed genes, ketoconazole, *Malassezia pachydermatis*, RNA-sequencing, stress response

## Abstract

*Malassezia pachydermatis* is an opportunistic yeast associated with otitis externa and dermatitis in dogs. Despite reduced susceptibility to azole antifungals has increasingly been reported in *M. pachydermatis*, the molecular mechanisms underlying azole stress responses in this species remain poorly understood. In this study, we investigated the global transcriptional response of two *M. pachydermatis* isolates with different ketoconazole minimum inhibitory concentration values following short-term ketoconazole exposure. Ketoconazole treatment resulted in slightly distinct transcriptional profiles in the two isolates. In isolate 12372, 80 genes were significantly upregulated and 120 were downregulated, whereas isolate 12693 showed 158 upregulated and 211 downregulated genes. Both isolates exhibited significant upregulation of genes involved in ergosterol biosynthesis, including *ERG3*, *ERG5*, *ERG11*, *ERG24*, and *ERG25* suggesting a conserved compensatory response to azole-mediated disruption of sterol metabolism. In isolate 12372, genes associated with ribosome biogenesis was predominantly downregulated, indicating reduced protein synthesis and growth-related activity. In isolate 12693, ketoconazole primarily suppressed genes involved in amino acid biosynthesis, and central carbon metabolism; and upregulated genes associated with ubiquitin ligases, RNA polymerase II regulators, and RNA-binding proteins. Notably, genes encoding ABC transporter-associated efflux pumps were not transcriptionally induced under the tested conditions. Our results provide a comprehensive transcriptomic profile of *M. pachydermatis* under azole stress, demonstrating that the yeast employs diverse metabolic strategies to enter a decelerated growth state and offer a good basis for identifying future therapeutic targets to combat azole resistance.

## 1 Introduction

*Malassezia pachydermatis* is a lipophilic yeast that can be found as a part of the microbiome of healthy skin and mucous membranes of domestic animals. However, due to environmental and endogenous conditions, *M. pachydermatis* can switch from commensal to pathogenic state, causing infections (1). The overgrowth of this yeast plays a significant role in the development of otitis externa and dermatitis in dogs. Treatment of *Malassezia* infections in dogs includes topical and/or systemic azole derivatives such as clotrimazole (CLT), itraconazole (ITR), ketoconazole (KTZ) and miconazole (MCZ). Antifungal therapies are usually effective in managing yeast overgrowth; nevertheless, treatment failure or rapid recurrence are frequently observed (2). The emergence of azole resistance has been reported in isolates from canine skin and ear canal worldwide (3–6). Fungal resistance to azole antifungals is primarily mediated by three distinct mechanisms: structural alterations in the ergosterol biosynthesis pathway, such as point mutations in the target enzyme lanosterol 14α-demethylase that reduce drug-binding affinity; upregulation of *ERG11*; or active efflux, mediated by overexpression of ATP-binding cassette (ABC) transporters (Cdr1/Cdr2) or major facilitator superfamily proteins (Mdr1) (7). Evaluation of resistance in *M. pachydermatis* is complicated, as a standardized methodology for determining the *in vitro* antifungal susceptibility profile of this opportunistic pathogen remains unestablished, and knowledge on molecular mechanisms underlying resistance is scant. Some amino acid substitutions in Erg11 have been proven to be associated with decreased susceptibility to different azoles. On the other hand, these substitutions did not confer pan-azole resistance, suggesting that multifactorial mechanisms may drive drug resistance (6, 8, 9). A comprehensive understanding of the global transcriptional response to antifungal agents may elucidate these underlying mechanisms and is crucial for identifying functional genomic elements and patterns of differential gene expression that govern cellular phenotypes. Currently, no data are available regarding the transcriptional response to azole stress in *M. pachydermatis* or the role of transcriptional changes in antifungal drug resistance. Therefore, the aim of our pilot study was to investigate genome-wide gene expression changes in *M. pachydermatis* isolates after KTZ treatment using transcriptome sequencing to identify affected biological pathways.

## 2 Materials and methods

*M. pachydermatis* isolates were gained from Duo-Bakt Veterinary Microbiology Laboratory. Isolates were originated from the ear canal of a bulldog (isolate 12372) and a Bernese Mountain Dog (isolate 12693) diagnosed with otitis externa. Previous antifungal susceptibility testing of these isolates showed minimum inhibitory concentration (MIC) values of 0.06 mg/L and 0.25 mg/L for KTZ; 0.06 mg/L and 0.5 mg/L for terbinafine; 4 mg/L and >32 mg/L for CLT; 0.5 mg/L and 16 mg/L for MCZ; and 0.125 mg/L and 0.125 mg/L for ITR, respectively (10). KTZ was chosen for further analysis since both oral and topical formulations are available (CLT and MCZ are used only topically), and it showed different MIC values during susceptibility testing. Isolates were cultured on Sabouraud dextrose agar (SDA) and incubated at 33 °C for 72 h. The initial concentration of fungal cell suspension in sterile saline was adjusted to an optical density of 1 McFarland, then a 1:10 dilution was carried out with Sabouraud dextrose broth (SDB) supplemented with chloramphenicol and 1% Tween 80 in a final volume of 20 mL. The suspension was incubated at 33 °C with shaking at 140 rpm for 4 h. After 4 h of pre-incubation, 0.03 mg/L KTZ in SDB was added to the treated cultures. This sub-MIC concentration of KTZ (0.03 mg/L) was selected to induce significantly affected growth without exerting lethal pressure on isolates, while the pharmacological effect was maintained. An equivalent volume of SDB was added to the untreated cultures to serve as a control. The cultures were then incubated for an additional 4 h with the abovementioned conditions. Experiments were carried out with two biological replicates. Aliquots of 100 μL were removed at 4 h after KTZ treatment from both treated and untreated samples, serially diluted 10-fold, and plated (100 μL) onto a single SDA plate. The plates were incubated at 33 °C for 72h, then viable cell count (CFU/mL) was determined to confirm the reduction in the cell count following KTZ treatment. For the transcriptome analysis studies, the yeasts grown in SDB were then centrifuged and the supernatant was discarded. Fungal cells were re-suspended on sterile phosphate-buffered saline and stored overnight at −70 °C before RNA extraction. Total RNA was extracted from treated and untreated cells of both isolates with two biological replicates using the Qiagen RNeasy Plant Mini Kit according to the manufacturer’s instructions. The concentration and quality of the obtained RNA were assessed using a NanoDrop spectrophotometer (Thermo Fisher Scientific, Wilmington, USA). Library preparation, mRNA sequencing on Illumina NovaSeq X Plus platform and data analysis were performed by Novogene Co., Ltd. (Cambridge, United Kingdom). Briefly, the generated paired-end reads were quality controlled and mapped to the reference genome (*M. pachydermatis* CBS 1879) with HISAT2 v2.2.1. DESeq2 algorithm v1.42.0 was used to obtain normalized gene expression values based on a negative binomial generalized linear model. Differentially expressed genes (DEGs) were identified using Benjamini–Hochberg false discovery rate (FDR) correction for multiple testing. Upregulated and downregulated genes were defined as DEGs based on the following criteria: log_2_ fold change ≥1 or ≤ − 1 and padj (adjusted *p*-value) ≤ 0.05. The clusterProfiler package version 4.8.1 was used for gene enrichment analysis, including the Gene Ontology (GO) and Kyoto Encyclopaedia of Genes and Genomes (KEGG) (significant enrichment: padj ≤ 0.05). The application of a stringent *p*-value correction (Benjamini–Hochberg FDR) in conjunction with a rigorous log_2_ fold change threshold ensured that only the most robustly DEGs were identified.

## 3 Results

**Figure 1:**
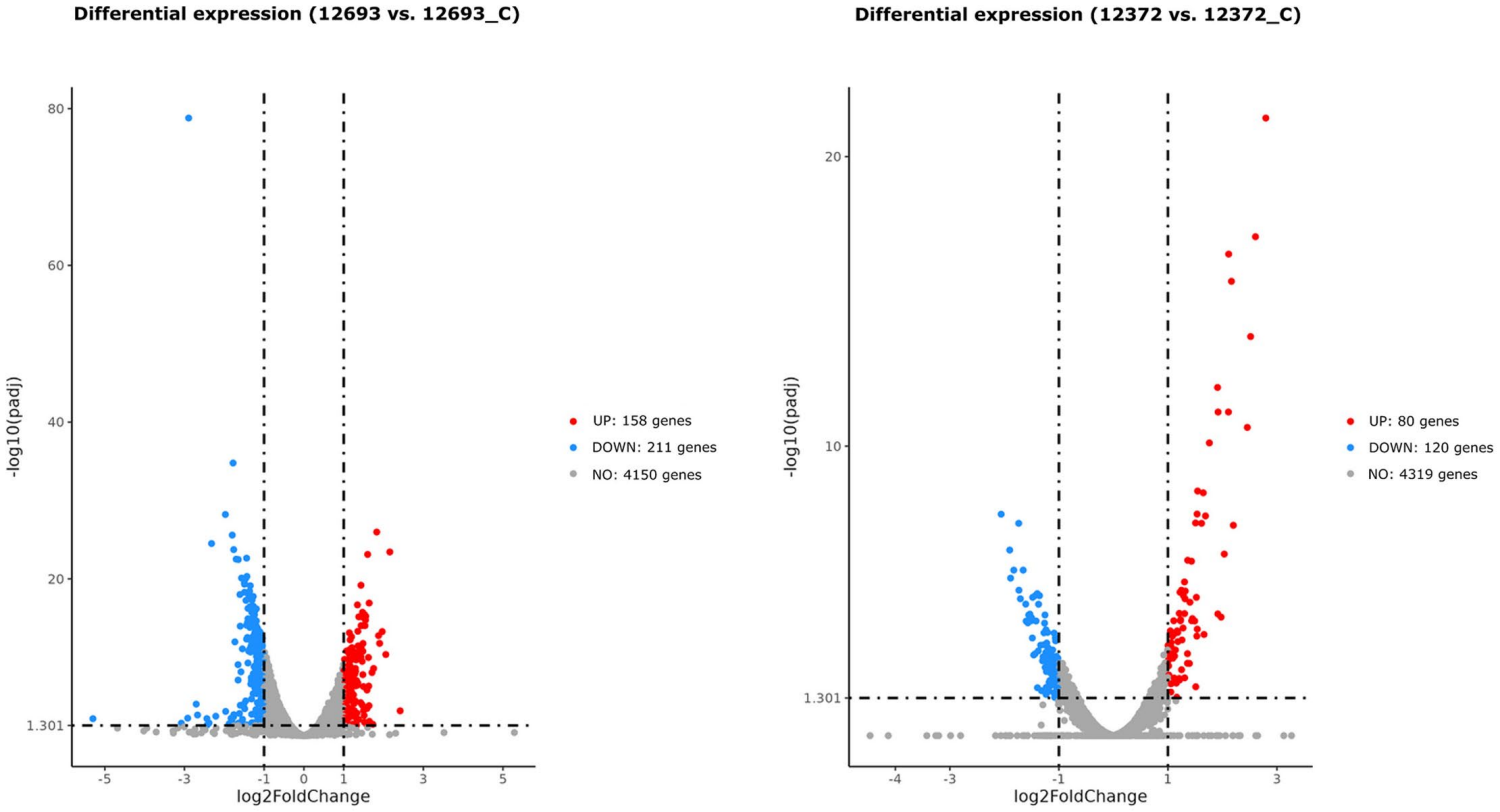
Volcano plot of differentially expressed genes identified in isolates 12693 and 12372 compared to untreated control in response to ketoconazole treatment. The horizontal dashed line indicates the threshold for differential gene screening criteria. Upregulated (red dot) and downregulated (blue dot) genes were defined as differentially expressed genes (DEGs) based on the following criteria: *log_2_* fold change ≥ 1 or ≤ − 1 and adjusted *p*-value (*padj*) ≤ 0.05. Gray dots indicate genes that did not meet the cutoff value (no significant changes). Full annotated DEG lists for both isolates are available in Supplementary material.

**Figure 2:**
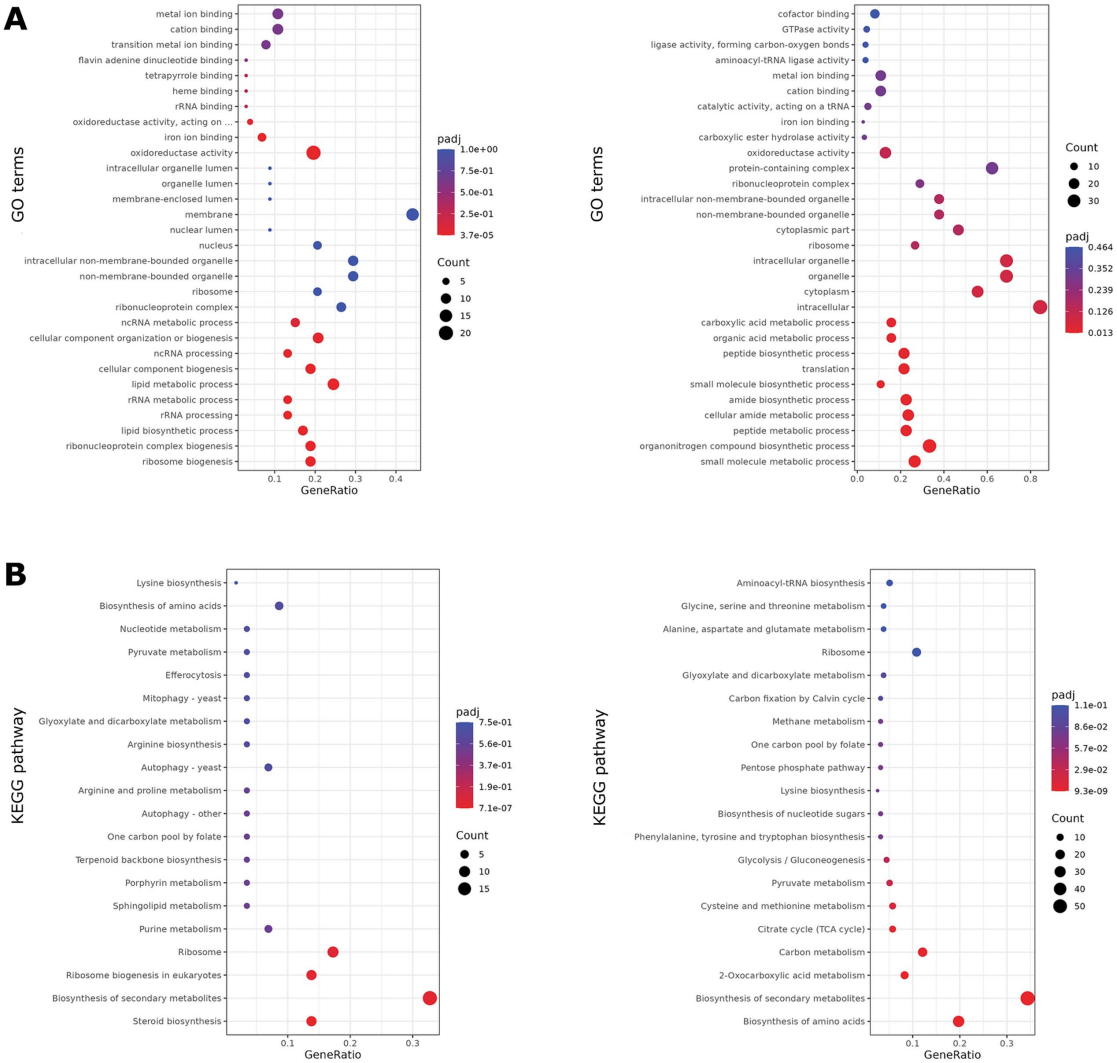
Enrichment analysis scatter plot of differentially expressed genes identified in isolates 12372 (left) and 12693 (right) using Gene Ontology **(A)** and Kyoto Encyclopaedia Genes and Genomes **(B)**. The figure represents the ratio of the differential gene number to the total number of differential genes on GO terms and KEGG pathways. The number of enriched genes is shown as the size of the dot, and the adjusted *p*-value is shown as the color shade of the dot.

KTZ treatment affected the growth of 12372 compared with the untreated cells (7.3–8.2× 10^6^ vs. 1.05–1.22×10^7^ CFU/mL), while the growth of 12693 was not inhibited (6.9–7.3×10^6^ vs. 6.5–6.8×10^6^ CFU/mL). By analyzing the transcriptional response to KTZ in different *M. pachydermatis* isolates, we observed 80 and 158 upregulated genes, while 120 and 211 genes were identified as being significantly downregulated in KTZ-treated “low MIC value” (12372) and “high MIC value” (12693) isolates compared to the untreated controls (Figure 1). GO enrichment analysis yielded five most enriched functional categories of GO terms in isolate 12372: ribosome biogenesis, ribonucleoprotein complex biogenesis, lipid biosynthetic process, rRNA processing and rRNA metabolic process. KEGG enrichment analysis revealed that genes were enriched across five pathways: steroid biosynthesis, biosynthesis of secondary metabolites, ribosome biogenesis, ribosome, and purine metabolism (Figure 2; Supplementary material). In 12693, the main enriched GO categories were small molecule metabolic process, organonitrogen compound biosynthetic process, peptide metabolic process, cellular amide metabolic process, and amide biosynthetic process. KEGG pathway analysis identified DEGs that are involved in biosynthesis of amino acids, biosynthesis of secondary metabolites, 2-Oxocarboxylic acid metabolism, carbon metabolism, citrate cycle (TCA cycle) (Figure 2; Supplementary material). Overall, no genes were identified that significantly upregulated in one isolate and downregulated in the other. In general, genes associated with ergosterol biosynthesis were significantly enriched among the upregulated gene sets in both treated isolates. In 12693, *ERG3* (C-5 sterol desaturase), *ERG5* (C-22 sterol desaturase), *ERG11* (lanosterol 14-alpha-demethylase), *ERG24* (C-14 sterol reductase) and *ERG25* (C-4 sterol methyl oxidase) was significantly upregulated, while in 12372, besides these *ERG* genes, *ERG1* (squalene epoxidase), *ERG2* (C-8 sterol isomerase), *ERG4* (C-24 (28) sterol reductase), *ERG6* (delta(24)-sterol C-methyltransferase), and *ERG20* (farnesyl pyrophosphate synthetase) were found to be significantly upregulated as well. Upregulated genes shared among isolates included genes encoding proteins responsible for transport and autophagy (lysosomal membrane GTPase, ubiquitin-like protein Atg8, E2-like enzyme Atg3, phospholipid transport protein, oligopeptide transmembrane transporter, NADH-cytochrome b5 reductase Cbr1); DNA repair and recombination (ssDNA endonuclease and repair protein Rad10, meiotic sister-chromatid recombination aldehyde dehydrogenase); signaling (sphingolipid delta-4 desaturase); protein folding and oxidative stress (endoplasmic oxidoreductin-1, NADH-cytochrome b5 reductase Cbr1), polyamine biosynthesis (agmatinase). Uniquely upregulated genes in 12693 is comprised of multiple ubiquitin ligases, RNA polymerase II regulators, and RNA-binding proteins, and lipid metabolism regulatory protein, while 12372 showed a targeted response mainly through ergosterol biosynthesis pathway to maintain membrane structure. The common downregulated gene sets of the treated isolates were primarily enriched for functions related to ribosomal proteins (60S and 40S ribosomal protein subunits such as L3, L4B, L7, L13, S8A), translation factors (eIF3, eIF4A, translation initiation factor 3 subunit b, EF-1α, EF-2), and ribosome biogenesis or ribosomal RNA processing (Nop7, Rpf2, small subunit processome components, snoRNA-associated proteins). Additionally, genes involved in amino acid and nucleotide biosynthesis (homocitrate synthase Lys21, glycine hydroxymethyltransferase Shm1, GMP synthase, inosine-5′-monophosphate dehydrogenase Imd1, amidophosphoribosyltransferase Ade4) were significantly downregulated. However, the rate of downregulated genes was much higher in 12692 than in 12372. In addition, genes downregulated only in 12693 include multiple mitochondrial respiratory chain complex I subunits and genes involved in carbohydrate metabolism (e.g., NAD-dependent isocitrate dehydrogenase Idh2, aconitate hydratase Aco1, glucose-6-phosphate isomerase Pgi1, phosphoglucomutase-2 Pgm2, malate dehydrogenase Mdh1, glycerone kinase). These transcriptomic changes indicate a more extensive reprogramming of transcription and protein homeostasis in 12693 following KTZ treatment relative to the response in 12372. Interestingly, no alteration in the expression of gene encoding efflux pumps, namely *ATM1*, was observed in the investigated isolates compared to the untreated controls.

## 4 Discussion

Fungi exhibit a remarkable capacity for adaptation to azole-induced stress through global transcriptional modification. The overexpression of genes encoding azole efflux pumps or target enzymes in ergosterol biosynthetic pathway represents the main mechanism underlying the decreased susceptibility observed in clinical isolates. Consequently, identifying the regulatory pathways governing transcriptional responses to azoles might provide potential targets for new therapeutic agents (11). In *Candida albicans*, several transcription factors have been identified that play a significant role in regulatory mechanisms in response to azoles. Tac1 regulates the expression of the ABC transporters Cdr1 and Cdr2, both of which function as azole efflux pumps. Furthermore, *C. albicans* has an additional azole efflux pump, Mdr1, a member of the major facilitator superfamily. The expression of Mdr1 is controlled by the transcription factor Mrr1. Some gain-of-function mutations in Mrr1 have been shown to cause *MDR1* overexpression that confers multidrug resistance (12–14). In *Saccharomyces cerevisiae*, the azole efflux pump Pdr5 (a homolog of Cdr1 and Cdr2) is transcriptionally regulated by the zinc-cluster transcription factors Pdr1 and the paralogue Pdr3. Interestingly, the expression of these transcription factors can be induced by chemically different drugs, including KTZ, cycloheximide and rifampicin (15). In *M. restricta* and *M. furfur*, the upregulation of *PDR5* and *PDR10* also contributes to azole resistance (16, 17). However, *in vitro* studies demonstrated that while the efflux pump inhibitors haloperidol, promethazine, and tacrolimus failed to enhance the susceptibility of *Malassezia* spp. to fluconazole, they significantly increased the activity of ITR, KTZ, and voriconazole. This observation suggests that resistance mechanisms against fluconazole could differ from those of other azoles in *Malassezia* species (18, 19). Regulation of the azole target gene *ERG11* is mediated by the transcription factor Upc2 in both *C. albicans* and *S. cerevisiae*. Notably, in azole-resistant clinical isolates of *C. albicans*, the overexpression of *ERG11* is predominantly attributed to gain-of-function mutations in *UPC2* (20, 21). In contrast, the *UPC2* or its homolog has not yet been detected in *Malassezia* spp.

While the mechanisms governing antifungal resistance in *Candida* species and human-pathogenic *Malassezia* species have been extensively investigated, the molecular basis of antifungal resistance in *M. pachydermatis* is yet to be fully elucidated. To the best of our knowledge, this is the first study evaluating the transcriptional changes in *M. pachydermatis* isolates under KTZ exposure. A similar pattern of upregulated genes was observed across isolates with varying KTZ MIC values. Enrichment analysis showed the upregulation of genes involved in ergosterol biosynthesis, such as *ERG3*, *ERG5*, *ERG11*, *ERG24* and *ERG25*. This reaction is consistent with general fungal stress response to the mechanism of action of azoles, as previously documented in other fungal species (22–24). This observation supports the biological relevance of our transcriptome sequencing data. Azole antifungals disrupt ergosterol biosynthesis via the inhibition of lanosterol 14-*α*-demethylase. This inhibition typically results in the depletion of membrane ergosterol alongside the concomitant accumulation of alternative sterol intermediates, including lanosterol, eburicol, and the cytotoxic 14-α-methyl-3,6-diol. Strict regulation of ergosterol homeostasis is crucial not only for maintaining ergosterol biosynthesis but also for preserving vital cell membrane functions (12). Therefore, overexpression of *ERG* genes may represent a compensatory mechanism in response to ergosterol depletion. Of note, slight differences were observed between the two isolates at transcriptional level. While isolate 12372 primarily upregulated the expression of *ERG* genes, isolate 12693 exhibited a broader stress response following KTZ treatment. Azoles perturb cell membrane integrity and compromises protein folding within the endoplasmic reticulum (ER) (25). In 12693, the upregulation of the Hrd1 ubiquitin ligase—a core component of the ER-associated degradation (ERAD) pathway—serves as a direct indicator of ER stress, facilitating the targeting and subsequent degradation of misfolded membrane and secretory proteins generated by this disruption. Furthermore, the broader induction of ubiquitin ligases (such ubiquitin-protein ligase Anaphase Promoting Complex) and autophagy (Atg8) reflects a systemic proteostatic response, wherein the cell clears damaged proteins and recycles cellular constituents to sustain localized membrane-remodeling processes.

Regarding azole-induced stress in fungi, simultaneous transcriptional changes of genes encoding enzymes in ergosterol biosynthesis and efflux pumps have been observed indicating that these pathways are activated by azoles (26, 27). Nevertheless, Hu et al. demonstrated that the transcriptional activation of the Cdr4 efflux pump in *Neurospora crassa* is correlated with the accumulation of specific sterol intermediates rather than the depletion of ergosterol (12). Hence, experimental data from other fungi are needed to explain the specific mechanisms driving transcriptional changes under azole stress. In our previous study (10), although ABC transporter genes were identified in the genomes of *M. pachydermatis* isolates, the present transcriptome analysis did not reveal upregulation of these genes under the tested conditions. Moreover, the expression of gene encoding ABC transporter ATP-binding protein (Arb1) was downregulated in both treated isolates. However, Arb1 might play a role in stimulating 40S and 60S ribosomal subunit biogenesis, rather than function as an efflux pump (28). The lack of ABC transporter induction suggests that, under the tested conditions, efflux pump overexpression was not a major component of the transcriptional response to KTZ. Based on these observations and previous susceptibility testing results, these isolates might be considered susceptible to KTZ (10).

Overall, multiple genes associated with amino acid biosynthesis, ribosome biogenesis, and carbohydrate metabolism were significantly downregulated in response to KTZ, although some differences between isolates 12372 and 12693 were noted. In isolate 12372, genes mostly involved in ribosome biogenesis were downregulated, including those involved in the synthesis, maturation, and assembly of rRNAs and ribosomal proteins. Ribosome biogenesis is a highly energy-consuming process (29). In response to KTZ, the *Malassezia* cells reduced their protein synthesis capacity and entered a decelerated growth state, which is consistent with the fungistatic activity of azoles. Of note, the nucleolar GTP-binding protein Nog1 and mRNA-binding biosynthesis protein Nop4 were also downregulated upon KTZ treatment. In eukaryotes, a complex containing 60S ribosomal proteins and the pre-ribosomal factors Nop7 and Rlp24 is formed during ribosome biosynthesis that is mediated by Nog1. Honma et al. (29) demonstrated that the Nog1-containing complex is immobilized in the nucleolus due to nutrient starvation or TOR (protein kinase, target of rapamycin) inactivation, resulting in the inhibition of pre-60S complex transport from the nucleus to the cytoplasm in *S. cerevisiae*. These findings underscore the critical role of the Nog1 complex in modulating ribosome biogenesis depending on nutrient availability. Our results showed that KTZ induces transcriptional alterations similar to nutrient depleted conditions as *NOG1, NOP4* and *NOP7* was downregulated, which further contribute to the inhibition of ribosome biosynthesis and cell proliferation.

In isolate 12693, the expression of a specific subset of genes involved in amino acid biosynthesis and central carbon metabolism was decreased along with genes involved in ribosome biogenesis. A similar metabolic response was observed in *C. albicans* to fluconazole treatment. Amino acid biosynthesis (e.g., glycine, proline, tryptophan, asparagine) was significantly reduced by 0.5–0.75-fold (30). Other transcript profiling studies revealed transcriptional changes under certain stress conditions, including repression of glycolysis and induction of glyoxylate cycle, *β*-oxidation, and gluconeogenesis (31, 32). The glycolytic pathway is strongly correlated with amino acid biosynthesis. In *C. albicans*, amino acid production relies on two main pathways: glycolysis generates precursors for almost all amino acid families, while the TCA cycle yields precursors for the aspartate and glutamate amino acid (33). In our study, the downregulation of genes that play a role in both metabolic processes (*ENO1*, *FBA1*, *PGI1*, *PGM2*, *ACO1*, *IDH2*, *MDH1*) was noticed in *M. pachydermatis*. These findings may indicate that the azole-induced stress prompts isolate 12693 to shift toward reduced anabolic state (even at low KTZ concentration), while reallocating carbon and reducing power to maintain membrane biosynthesis.

In conclusion, the two *M. pachydermatis* isolates displayed partially distinct transcriptional responses to short-term KTZ exposure. Both isolates showed induction of ergosterol biosynthesis genes, whereas main downregulated pathways differed between isolates, involving ribosome biogenesis in isolate 12372 and ribosome biogenesis, amino acid biosynthesis and central carbon metabolism in isolate 12693. This rapid (4 h post-treatment), extensive transcriptional response of isolate 12693 to the KTZ-stress may give a possible explanation for the sustained cell viability and the higher KTZ MIC value compared to isolate 12372. Although the present pilot study is limited by the small sample size (*n* = 2), a single KTZ concentration, and the absence of independent qPCR validation, the strong internal consistency of the RNA-Seq datasets supports the robustness of the observed transcriptional patterns. To our knowledge, this study provides the first comprehensive transcriptome-wide characterization of azole stress responses in *M. pachydermatis*. As such, it addresses an important knowledge gap in veterinary mycology and establishes a good basis for future investigations incorporating larger isolate collections, resistant phenotypes, metabolomic analyses, and functional genomics approaches. These studies will be essential to determine whether the identified transcriptional patterns are associated with antifungal susceptibility, to elucidate the molecular mechanisms underlying azole adaptation and tolerance, and to identify potential targets for the development of novel antifungal strategies.

## Data Availability

Raw reads obtained during the current study are available at the NCBI Sequence Read Archive under BioProject PRJNA1473253.
